# Alluvial connectivity in multi‐channel networks in rivers and estuaries

**DOI:** 10.1002/esp.5261

**Published:** 2021-12-07

**Authors:** Willem Sonke, Maarten G. Kleinhans, Bettina Speckmann, Wout M. van Dijk, Matthew Hiatt

**Affiliations:** ^1^ TU Eindhoven Eindhoven The Netherlands; ^2^ Faculty of Geosciences Utrecht University Utrecht The Netherlands; ^3^ Arcadis, Water and Environment Division Zwolle The Netherlands; ^4^ Rijkswaterstaat Zee and Delta, Netwerk Ontwikkeling and Visie Division Rijswijk The Netherlands; ^5^ Department of Oceanography and Coastal Sciences Louisiana State University Baton Rouge Louisiana USA

**Keywords:** algorithms, bifurcation, connectivity, estuary, network

## Abstract

Channels in rivers and estuaries are the main paths of fluvial and tidal currents that transport sediment through the system. While network representations of multi‐channel systems and their connectivity are quite useful for characterisation of braiding patterns and dynamics, the recognition of channels and their properties is complicated because of the large bed elevation variations, such as shallow shoals and bed steps that render channels visually disconnected. We present and analyse two mathematically rigorous methods to identify channel networks from a terrain model of the river bed. Both methods construct a dense network of locally steepest‐descent channels from saddle points on the terrain, and select a subset of channels with a certain minimum sediment volume between them. This is closely linked to the main mechanism of channel formation and change by displacement of sediment volume. The two methods differ in how they compute these sediment volumes: either globally through the entire length of the river, or locally. We compare the methods for the measured bathymetry of the Western Scheldt estuary, The Netherlands, over the past decades. The global method is overly sensitive to small changes elsewhere in the network compared to the local method. We conclude that the local method works best conceptually and for stability reasons. The associated concept of alluvial connectivity between channels in a network is thus the inverse of the volume of sediment that must be displaced to merge the channels. Our method opens up possibilities for new analyses as shown in two examples. First, it shows a clear pattern of scale dependence on volume of the total network length and of the number of nodes by a power law relation, showing that the smaller channels are relatively much shorter. Second, channel bifurcations were found to be predominantly mildly asymmetrical, which is unexpected from fluvial bifurcation theory.

## Graphical abstract

Two novel, mathematically rigorous algorithms to identify channel networks in bathymetries of fluvial systems were compared conceptually and empirically on a dataset of the Western Scheldt estuary. Alluvial connectivity is related to the sediment volume that needs to be eroded for two tidal channels to merge.

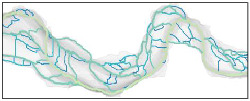

## 1 INTRODUCTION

Rivers and estuaries are important natural landscapes that enable agriculture and transportation of goods, but they also flood and shift their course, with dire consequences for all that live around them. Many aspects of the functioning of rivers and estuaries depend on the connectivity of channels (Wohl et al., 2019). The analysis and prediction of the complex behaviour of channel networks in fluvial systems is relevant in view of transportation, valuable habitats and coastal flood hazards including future sea‐level rise. Network dynamics have significant consequences for the hydromorphological and ecological functioning of river systems and, consequently, for human society (Best, 2019; van Dijk et al., 2019). For instance, shipping lane deepening by removal of shallow areas in the Western Scheldt main channel caused deeper tidal penetration, which presently increases flood risk in Antwerp, which in turn is mitigated by costly measures.

**FIGURE 1:**
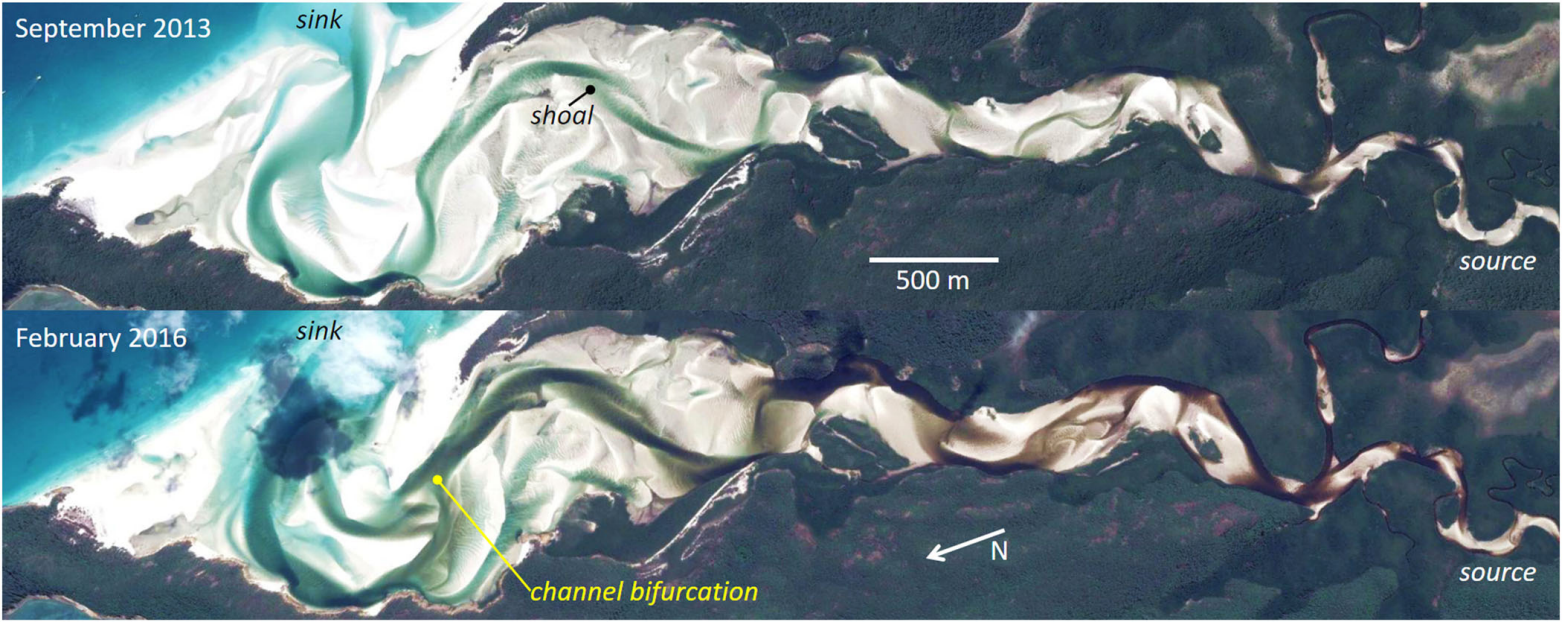
Whitehaven estuary (Australia, 20° 16^*′*^ S, 149° 1^*′*^ E). Both images show channel segments and dead‐ended channels disconnected from the network by shoals and bars. The 2013 image was taken at a slightly lower water surface elevation than the 2016 image. Image source: Google Earth, accessed March 2021 [Color figure can be viewed at wileyonlinelibrary.com]

The multiple channels in braided rivers, deltas and river mouths are key elements that determine network properties, dynamics and development (Howard et al., 1970; Kleinhans et al., 2013). The bars between these channels range in length from about one water depth to ten times the overall river width (Hicks et al., 2002; Parker, 1976). Network analysis is potentially a powerful tool to study the complicated patterns and to answer fundamental questions about morphodynamics of nodes, or bifurcations and confluences (Kleinhans et al., 2013), and network properties that emerge as a result of underlying morphodynamic processes (Marra et al., 2014; Passalacqua et al., 2013; Phillips et al., 2015; Tejedor et al., 2015a, 2015b). These properties have morphological meaning and may be informative of the mechanisms that form and change channel patterns in the world. For example, braided rivers evolve merging and splitting channels under the influence of local water‐level gradients caused by floods, and the resulting sediment transport that leads to channel cutting and bar development (e.g., Marra et al., 2014; Schuurman et al., 2016). In estuaries, reversing currents due to tides add considerable complexity to the changing ‘braided’ network patterns (Figure 1) (Hiatt et al., 2020, 2021, this issue). While sediment transport cannot practically be measured in a great many locations in any system, the net result of morphological change can be captured with various methods in repeated bed elevation mapping. The rationale to reduce a continuous field, namely the bed surface of an aquatic system, to a channel network, is rooted in the physical processes of sand and gravel transport and of channel formation and channel dynamics, briefly reviewed below. In turn, the identification of networks from data must account for the channel pattern properties to capture the complexity.

The fundamental mechanism that leads to bar and channel pattern formation causes great variation in depth. Most sediment transport takes place in the channels, because sediment transport is a nonlinear function of the flow depth and flow velocity. The underlying causes for this nonlinearity are the higher density of sediment compared to water, causing a non‐zero asymptote of flow shear stress above which sediment is mobilised and below which it is immobile, and the friction between the particles as well as the depth‐related friction of the flow, all of which are incorporated into sediment transport functions (e.g., Soulsby & Damgaard, 2005). The effect of the nonlinearity is a tendency of growing perturbations with accreting bars and deepening channels: a slightly deeper patch in an otherwise uniform channel will have stronger flow, a higher bed shear stress and a disproportionally higher sediment transport rate. This leads to further deepening, because upstream the transport rate is not enlarged. This fundamental and basic instability in the water–sediment–bed system causes channels and bars with channel bifurcations in all systems with flow over granular material on Earth, Mars and Titan (Parker, 1976, and many similar theories developed since). The deepening is counteracted by gravity‐driven sediment motion on slopes, which get steeper as channels become deeper (Baar et al., 2019; Parker, 1976). Ultimately the balance between the channel carving and the slope processes determines channel width–depth ratio, bar dimensions (Schramkowski et al., 2002; Struiksma et al., 1985) and channel bifurcation instability leading to avulsion (Bolla Pittaluga et al., 2015), and underlies theories for fluvial and tidal bars (Leuven et al., 2016; Schramkowski et al., 2002; Struiksma et al., 1985) and for bifurcations (Bolla Pittaluga et al., 2015; Kleinhans et al., 2013) as well as numerical morphological models (Baar et al., 2019; Schuurman et al., 2013). In other words, channels with great variation in depth are intrinsic features of rivers, estuaries and deltas.

During flood events and high tide, multiple channels convey flow and have sediment transport, but this does not mean that the channels can be defined by a steepest descent path on the bed surface (Kleinhans et al., 2017, 2019; Limaye, 2017). Instead, the bed surface of a multi‐channel system is better characterised by a series of pools separated by usually submerged shoals. This is similar but subtly different between braided rivers and ‘braided’, multi‐channel estuaries. In braided rivers, the thalweg and parallel channel paths vary greatly in depth. As a result, the number of channels in the network varies strongly with discharge, with an increasing number of flooded channels during increasing discharge but a decreasing number during higher discharge as bars between channels flood. This means that additional information or modelling of the flow field would be needed to establish the flow network (Kleinhans et al., 2017, 2019; Limaye, 2017). Estuaries exhibit similar properties to braided rivers, but with unique and enigmatic channel configurations such as the ‘mutually evasive ebb‐ and flood‐channels’ (Leuven et al., 2016), where the landward end of a flood channel is separated by a shoal from the seaward end of an ebb channel, rather than be connected without the shallow zone. Even the largest channels are surprisingly poorly connected due to the presence of shallower bars and shoals on one end or both ends, or between two adjacent channels (Figure 1). As in braided rivers, the water level in estuaries is also highly variable due to tides, river floods and storm surges. Regardless of the depth variation and the failure to identify channels through steep descent or separated flow paths, the channels are in reality connected through fluxes of water and sediment. In rivers with a non‐zero flow discharge there is at least one path in the network on which the channels are connected. In estuaries, the tide comes in and goes out over the shoals and bars.

It is without doubt that flow and sediment fluxes connect the seemingly (partly) disconnected channels. However, neither a simple depth threshold nor a steepest descent method would allow the *identification* of a network of connected pools as channels from bathymetric data (Hiatt et al., 2020; Kleinhans et al., 2017, 2019; Limaye, 2017). In addition to the depth variation elaborated above, water can fill up local minima in the river bed, such that water flows over these minima, causing the river bed to ascend instead of descend. Also, the inertia of flowing water can result in deviations from the true steepest descent direction. Finally, if a river always follows the steepest descent, it could never bifurcate, because each point in the digital elevation model (DEM) generally only has a single direction of steepest descent. All these issues mean that simple methods for channel identification on bathymetry fail to represent the inherent connectivity. While we can digitise channel networks on imagery, fundamental questions about network character and dynamics remain as yet unanswered, because the computational tools are lacking to rigorously and objectively identify channel networks in rivers and estuaries and study the changes over time.

Through channel formation, migration and displacement, most of the morphological work takes place by the reworking of alluvial material. Channels and bars may split and merge in multiple ways. Channel splitting can occur through mid‐channel bar formation and by cutoffs through bars (Ashmore, 1991; Bertoldi et al., 2009; Schuurman et al., 2016; Swinkels et al., 2009). The bar‐cutting channels may sweep over the bar or even remove it (Leuven et al., 2018; Swinkels et al., 2009), while removal of bar‐cutting channels leads to increasing bar elevation, as shown by the effects of sediment disposal (van Dijk et al., 2021). Due to instability at bifurcations, channels may also fill to become abandoned (Ashmore, 1991; Bolla Pittaluga et al., 2015; Kleinhans et al., 2013). Channel merging also occurs through lateral channel migration caused by local curvature (Ashmore, 1991; Leuven et al., 2018; Schuurman et al., 2013; Shimozono et al., 2019). Collectively, these perpetually dynamic processes ignite changes elsewhere through displacement of volumes of sand (Ashmore, 1991; Bertoldi et al., 2009). The notion that sediment volume displacement is central to the channel pattern is also supported by the effects of human interference: channel deepening, dredging and disposal of sediment reduce lateral channel migration and increased bar growth depending on the displaced sediment volume and location (van Dijk et al., 2021). As a result, many channels are somewhat connected across shoals or nearly connected, as only a limited volume would need to be removed for a higher connectivity, and (modelled) fluxes and a steepest descent method both fail to detect this kind of connectivity.

In general, the number of bathymetric datasets and models of continuously changing landscapes is growing explosively, but the tools to analyse them lag behind considerably. Regardless of the clear rationale for network representation of multi‐channel systems, geoscientists and engineers struggle with the creation and analysis of networks from spatial data such as satellite imagery and echo‐sounding bathymetries. In particular, there are currently no tools to connect sequential spatial networks in a mathematically rigorous way, while changes in their structure are frequent and large (Marra et al., 2014; van Dijk et al., 2019). Present practice relies on somewhat arbitrary rules to connect links and nodes through time in order to compare between networks. This lack of adequate algorithmic tools makes the study of the critical elements in the network, namely the channel bifurcation–confluence units (Kleinhans et al., 2013), and the emergent system behaviour almost impossible. Furthermore, without morphologically meaningful similarity measures and efficient algorithms to compute them, one cannot assess how similar networks produced by numerical models (e.g., Baar et al., 2019; van Dijk et al., 2019) and experiments (e.g., Braat et al., 2019; van Dijk et al., 2021) are to their counterparts in nature, which hence limits the potential of simulation for the prediction of the dynamic behaviour of river networks. Finally, the representation of large datasets in efficient networks with relevant characteristics potentially is a powerful data reduction tool. In short, a trustworthy automated approach for channel network detection would open up a myriad of possibilities to use the available big data of bathymetry.

In this paper, alluvial channel connectivity is conceptualised in the context of fluvial and estuarine morphodynamics, and is practically applied with a new tool on a large dataset of bathymetry. Here, alluvial connectivity is purely morphological, and alluvial connectivity should account for the fact that pools are to some degree connected across shoals and bars to form channels that convey water and sediment. The abstracted, morphological network is extracted from morphological data alone to avoid the dependence of network size and connectivity on the fast temporal hydrodynamics.

To this end, two different algorithms for mathematically rigorous channel network detection on bathymetries are presented and compared. Both methods use a DEM of the river bed as input and result in a network in which each channel is somehow ‘low’, and channels are separated by ‘sufficient’ sediment volume in longitudinal and lateral directions. The smaller this volume, the higher the connectivity, which takes account of shoals in somewhat connected channels and of situations with narrow bars between channels that are potentially laterally connected during high water levels or following channel cutting through the bar or removal of the bar by lateral channel migration. We automated the process of constructing channel networks, by modelling them based on a DEM of the river bed. While previous studies used an imagery‐based approach to detect presence/absence of channels, motivated by the high data availability worldwide, the water level in a river and the number of recognisable channels are highly variable, whereas river bed DEMs do not show this short‐term variability.

Both methods are here applied to the same dataset of 33 measured bathymetries of the Western Scheldt estuary collected between 1955 and 2015, at a decreasing time interval, by Rijkswaterstaat (of the Dutch Ministry of Infrastructure and Water Management, ‘vaklodingen’ data). The echosounding data was interpolated to yield maps at the same spatial resolution of 50 m for the entire period. The results are compared through quantification of total length of the network, total number of nodes and local bifurcation morphology. As the two algorithms compute the sediment volumes in fundamentally different ways, the conceptualisation of connectivity that the two kinds of networks entail is also different. Distinct behaviour is expected and will be discussed in view of the different methods and in terms of geomorphological meaning and use for multi‐channel systems.

## 2 METHODS: TWO CHANNEL NETWORK DETECTION ALGORITHMS

### 2.1 Earlier methods and present approach

In a river or estuary network, the links represent the channels, while the nodes represent places where these channels split (bifurcation) or merge (confluence), depending on the flow direction. In an estuary, due to the changing flow direction throughout the tidal cycle, a single node can represent a bifurcation in the ebb phase and a confluence in the flow phase, or vice versa. In computational geometry, algorithms for computing networks from high‐relief DEMs are well studied, for example, in the context of drainage networks or flows (Agarwal et al., 1996; Arge et al., 2003; de Berg et al., 2010; Phillips et al., 2015; Wohl et al., 2019; Yu et al., 1997). But most models for water flow in terrains, including drainage networks, generally assume that water follows the direction of steepest descent. On steep terrains this is a reasonable assumption, but channels within a braided river or estuary do not necessarily follow steepest descent in the river bed DEM (Hiatt et al., 2020; Kleinhans et al., 2019; Limaye, 2017). For the issues with morphological connectivity reviewed in the Introduction, the steepest descent direction does not satisfactorily model water flow in braided rivers and estuaries. Therefore, instead of steepest descent paths, the methods focus on finding low paths through the river bed, which may descend and ascend locally to connect deeper channels across shallower parts.

### 2.2 Topological background

**FIGURE 2:**
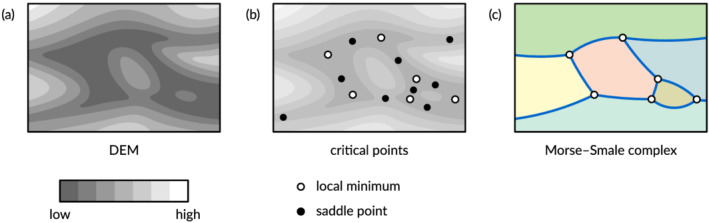
Sketch of a river‐bed DEM (a), minima and saddle points (b), and the Morse–Smale complex, with the Morse–Smale edges in dark blue and the Morse–Smale cells coloured (c) [Color figure can be viewed at wileyonlinelibrary.com]

To define which paths are considered low and to measure terrain volumes, we use a construct from topology called the *Morse–Smale complex* (Edelsbrunner et al., 2001). This complex contains all (local) minima and saddle points in the DEM, so‐called *critical points*, plus steepest‐descent paths, called *Morse–Smale edges*, from the saddle points down to the minima. The Morse–Smale edges divide the DEM into pieces, which are called *Morse–Smale cells* (Figure 2). The Morse–Smale complex of a river‐bed DEM is generally very dense, because measurement errors and small variations in the terrain generally cause a multitude of minima and saddles. We therefore need to select a subset of the Morse–Smale edges to form a morphologically meaningful river network.

The aim of both methods detailed below is to select Morse–Smale edges that form significant channels in the river. Here we define ‘significant’ in morphological terms, namely the volume of sediment involved. This concept is also intuitively close to how morphological change depends inversely on length scale, and how dredgers interfere in fluvial systems to improve navigability or augment sand for coastal defence. In rivers and estuaries where water levels that submerge most of the bathymetry occur frequently, two channels can be considered close if the volume of terrain between the channels is small. Relatively little morphological work is needed to remove a small volume of sediment by erosion that would merge the two channels into one. Therefore, we select paths that have enough volume of sediment between them.

Both methods start from the Morse–Smale complex and select paths based on sediment volumes, but differ in how they measure sediment volumes and select paths.

### 2.3 Striation (global) method

This method is based on the concept of a lowest path. Given two points *a* and *b* on the river bed, the lowest path between *a* and *b* is the path whose overall highest point is the lowest, breaking ties using the second highest point, then the third highest point, and so on. A lowest path generally passes through minima and saddle points in the DEM, and through pieces of Morse–Smale edges (i.e., steepest‐descent paths) between them. This can be understood intuitively as any other path in the neighbourhood would be higher than a steepest‐descent path from a saddle point. One can also prove this property formally (Kleinhans et al., 2019) by showing that the lowest path between two saddle points is always a part of the Morse–Smale complex.

**FIGURE 3:**
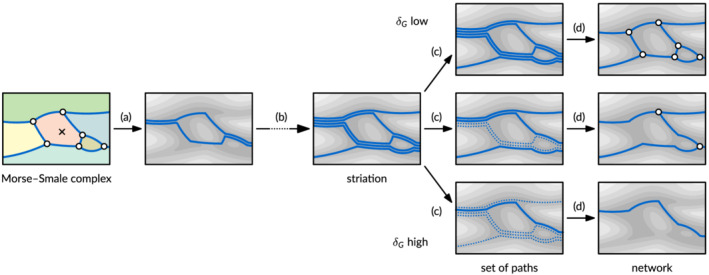
Steps in the striation method: (a) select a Morse–Smale cell (here marked with a cross) and compute the two lowest paths around it; (b) repeat this procedure until all Morse–Smale cells have been used; (c) select paths with volume at least *δ*_*G*_ between them (shown here for three values of *δ*_*G*_); (d) post‐process the selected paths into a network (figure adapted from Hiatt et al., 2020) [Color figure can be viewed at wileyonlinelibrary.com]

To generate a network (Figure 3), the method computes the lowest path through the river from the source to the sink, which splits the river into two pieces. In both pieces, we then keep repeating the same procedure, splitting the river until each piece contains only a single Morse–Smale cell. This results in a set of partially overlapping low paths through the DEM, which we call the *striation* of the river. Because we split the river along the lowest path, afterwards the lowest path through both pieces is on the boundary. As a result, if we repeat the lowest path computation recursively on the subpieces, we will not make any progress, staying on the same lowest path forever. To ensure we make progress, in practice we instead split off an entire Morse–Smale cell at a time (Figure 3a). Thus we add two paths to the striation at a time, namely the two lowest paths around the Morse–Smale cell.

The resulting striation contains many paths, so we need to select a subset of the striation paths that have enough volume between them (Figure 3c). Therefore, we need a method that, given two striation paths *p*_1_ and *p*_2_, computes the volume between them. This is challenging, because it is not immediately obvious which part of the terrain volume should be measured. Our solution is called the *sand function*, which is defined mathematically by a striation–monotone isotopy (Kleinhans et al., 2019). Informally stated, it measures the minimum volume of sand that needs to be removed for *p*_1_ to be able to slide downhill to *p*_2_, while still visiting the striation cells in order. Note that the sand function computes volumes globally over the entire length of the river in the DEM. That is, if two otherwise equal paths differ in two distinct places in the DEM, both places may contribute sediment volume to the sand function. This allows morphological interaction between far‐away parts of the river network.

We sort the striation paths on how low they are, starting with the overall lowest path. Then we select them one by one, as long as they are not too close to already selected paths. ‘Too close’ is measured by a user‐specified sand volume threshold *δ*_*G*_ (with *G* standing for global). The value of this parameter thus influences how many paths are included in the network, and thereby the scale or granularity of the network. Finally, the set of selected paths are post‐processed to form the final network (Figure 3d).

### 2.4 Persistence (local) method

The second method also starts from the Morse–Smale complex, but it computes sediment volumes locally, instead of taking the entire length of the river into account as in the striation method. The persistence method aims to find Morse–Smale cells (representing bars) containing at least sediment volume *δ*_*L*_ (with *L* standing for local). It achieves this by merging adjacent bars together until they have reached volume *δ*_*L*_.

This iterative merging of cells can be described by a general concept from topology, called *persistence*, which was introduced by Edelsbrunner et al. (2002). In the case of a DEM, persistence‐based simplification is similar to a simplification step based on topographic prominence, where all mountains with prominence below a certain threshold height are removed.

We extended the idea of persistence to use volumes instead of heights and call this *volume persistence*. In this case, we remove from the Morse–Smale complex of the river bed all Morse–Smale edges that separate bars that have volume smaller than *δ*_*L*_ in order to leave a single path. In a sense, this can be seen as stepwise DEM smoothing, or simplification, by shaving off these particular bars, but our analysis is done on the Morse–Smale complex and the smoothed DEM is not calculated. A similar method has previously been used (Carr et al., 2004) to simplify contour trees, which capture the topological structure of a 2D terrain, using so‐called local geometric measures, such as the line length of the contour, the area enclosed by the contour, or the volume of the enclosed region. This last type of persistence is exactly the one we use.

**FIGURE 4:**
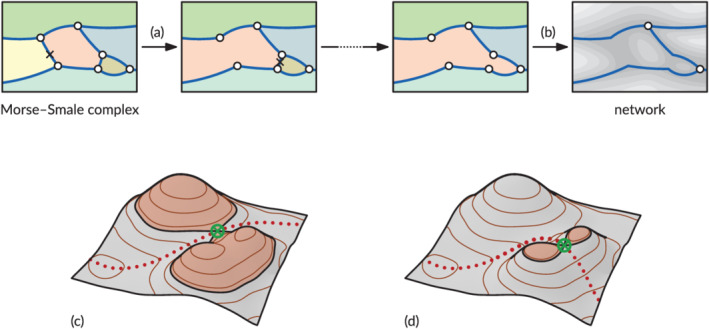
Steps in the volume persistence algorithm: repeatedly select a saddle in the Morse–Smale complex (a), and if the volume of sediment around the saddle is lower than the threshold remove the saddle to merge the adjacent Morse–Smale cells. After processing all saddles, the final network remains (b). (c,d) Two examples of computing volumes around saddles (the red‐shaded area indicates the measured volume) [Color figure can be viewed at wileyonlinelibrary.com]

To compute a network using volume persistence, we use the following algorithm. We first sort the saddle points in the Morse–Smale complex by height. Then, we compute for each saddle *s* (from high to low) how much volume of sediment exists in the terrain in the two Morse–Smale cells adjacent to *s*. This volume computation uses the height of *s* as a base; that is, we ‘cut off’ the terrain at the height of *s* and measure all the terrain volume above the cut surface (Figure 4). If a volume measured is smaller than *δ*_*L*_, we remove *s* and its Morse–Smale edges from the network, effectively merging the two adjacent Morse–Smale cells into one. After processing all saddles, the remaining Morse–Smale edges in our volume‐persistent terrain form significant channels.

The persistence method has the useful property that increasing the volume threshold *δ*_*L*_ will only cause more Morse–Smale cells to be merged. This means that for *δ*_*L*_ > *δ_L_′*, the channels in the network for *δ*_*L*_ are a subset of the channels in the network for *δ_L_′*. This property, which does not hold for the striation method, allows us to compute a single multi‐scale river network, where each channel is annotated with its own *δ*_*L*_ value.

### 2.5 Implementation

Both methods have been implemented in a freely available tool called TTGA (Topological Tools for Geomorphological Analysis). As input, TTGA reads a river‐bed DEM from a text or greyscale image file, plus a boundary that can be used to delineate the area of study. It then computes the Morse–Smale complex, and (striation method) the striation or (persistence method) the volume‐persistence simplification, and finally the river network. The resulting network can be viewed or exported for further analysis. TTGA supports a command‐line mode such that the creation of river networks can be scripted if desired. For the analyses in this paper, we used the command‐line mode to generate networks for all years fully automatically, and then analysed the resulting networks with custom scripts.

### 2.6 Analyses of the fluvial networks

The resulting networks are analysed in three ways. First, a map comparison of networks constructed with both methods allows a direct comparison of the correspondence in identified channel positions and of the volumes associated with the channels. This allows for a qualitative assessment of the differences between the methods.

Additionally, while the network methods were designed for bathymetry, they can potentially be applied to other fields. Flow velocity, in the context of estuaries, is an interesting field as the flow patterns may be different in the ebb and flood phase over the same bathymetry. As an experiment, a comparison is conducted between a bathymetry‐based network and networks calculated for two flow fields: that of the ebb and that of the flood phase, modelled with the operational flow model Simona used by Rijkswaterstaat. This will be a first qualitative test of the degree to which the morphological network is informative of the flow network, where flow direction is accounted for by having two maps of different flow phases. This analysis may show potential for future research on comparison of networks of different but causally connected properties in the same system.

Second, properties of the entire network are compared between the two methods: total network length of all the links below a volume threshold, total number of nodes, and the local, map‐based comparison between the channel networks of the two methods. This also allows for a quantitative characterisation of the network of the persistence method in the framework of network scaling. Here, a scale‐free network would be one of which the degree distribution follows a power law with an exponent between −2 and −3, meaning that the number of links decreases rapidly with increasing volume.

Third, we show how the network allows analyses of important morphological characteristics and elements of channel networks. Two key morphological characteristics of channel bifurcations are the horizontal angle between the bifurcating channels and the step in bed elevation, which have been studied in rivers but have so far barely been considered in estuaries. On the basis of observations and theory for rivers we expect that, where one of the bifurcated channels is wider and carries more flow than the other, the bed elevation in the smaller channel is higher and the angle with the upstream channel is also higher (Kleinhans et al., 2013). This was already supported by analysis with the striation method, where the smaller bifurcates were often oriented more perpendicular to the main flow direction in an idealised braided river model and an idealised estuary model (Hiatt et al., 2020).

**FIGURE 5:**
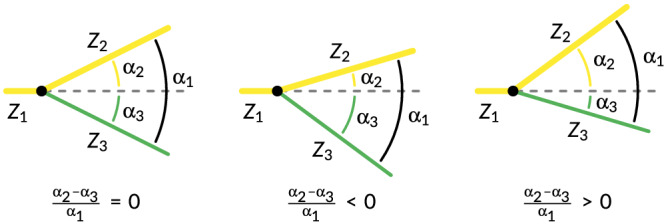
Definition and examples of bifurcation angle asymmetry [Color figure can be viewed at wileyonlinelibrary.com]

The persistence network for volumes larger than 10^5^ m^3^ was divided into classes to correspond to the channel taxonomy in use for the Western Scheldt: side channels are connected on both ends to the lowest path, which is the main channel, and the smaller connecting channels are connected on one or two ends to side channels. The networks allow the detection of bifurcations, defined as having one upstream channel and two downstream channels, and the extraction of geometric properties. Several attempts to track bifurcations and channels through time with search radii failed, so analysis of bifurcations through time was not further pursued. Here, upstream and downstream are defined in landward or seaward direction on the main channel, with the two bifurcates pointing in the opposite direction. Here, the angle between the bifurcates is smaller than the angle between either one of the bifurcates and the undivided channel. The undivided channel is numbered 1; the larger of the two bifurcates, defined in terms of persistence volume in the present analysis, has number 2; and the smallest channel has number 3 (Figure 5).

To compare the degree of bed elevation asymmetry of bifurcations of very different sizes, the bed elevation difference between the larger and smaller bifurcates (in terms of persistence volume) was normalised by the bed elevation of the upstream channel averaged over a length scale defined by channel width and standardised for automated analysis to 1 km for a connecting channel or 1.5 km for a side channel:

(1) $$Z_{n}=\frac{Z_{2}-Z_{3}}{Z_{1}},$$

where bed elevation is here given in metres above mean sea level (AMSL), meaning that *Z*_1, 2, 3_ are approximately the time‐averaged channel depths (as in an estuary, the water height is approximately 0 m AMSL). As such, the above equation normalises the depth difference between the bifurcates by the upstream channel depth. Here, *Z*_*n*_ = 0 indicates a vertically symmetric bifurcation without a bed elevation jump, *Z*_*n*_ < 0 indicates that the minor bifurcate is shallower and *Z*_*n*_ > 0 means a deeper minor bifurcate and values of *Z*_*n*_ ≈ 1 or *Z*_*n*_ ≈ −1 indicate extremely asymmetric bifurcations in terms of water depth or bed step.

Likewise, the angle asymmetry of the bifurcations was calculated as the difference between the angles of the smaller and larger bifurcates (in terms of persistence volume) with the upstream channel, measured over a length of one channel width, normalised by the sum of the two angles:

(2) $$\alpha_{n}=\frac{\alpha_{2}-\alpha_{3}}{\alpha_{1}},$$

where *α*_2, 3_ are the angles between the upstream channel and each bifurcate and $\alpha_{1}=|\alpha_{2}|+|\alpha_{3}|$ is the angle between the bifurcates (Figure 5). Here, *α*_*n*_ = 0 indicates a horizontally symmetric bifurcation, *α*_*n*_ < 0 means that the larger bifurcate is more aligned with the upstream channel, and *α*_*n*_ > 0 means that the smaller bifurcated channel is more aligned.

There are various ways to normalise the bed steps and changes in depth or cross‐sectional area at bifurcations, all of which are methods to reduce three‐dimensional data with a focus on particular aspects (Kleinhans et al., 2013). Here, with the water height at 0 m AMSL, the measure represents asymmetry of water depth and of the bed jump at the entry of the bifurcated channels, which ignores width asymmetry or flux asymmetry. Both latter asymmetries require more data; a width asymmetry would require the far from trivial identification of channel boundaries on the basis of bathymetry combined with the one‐dimensional network. For illustration of the potential of the network identification for analysis of bifurcations, the depth asymmetry and angle asymmetry suffice.

## 3 RESULTS

### 3.1 Map comparisons

**FIGURE 6:**
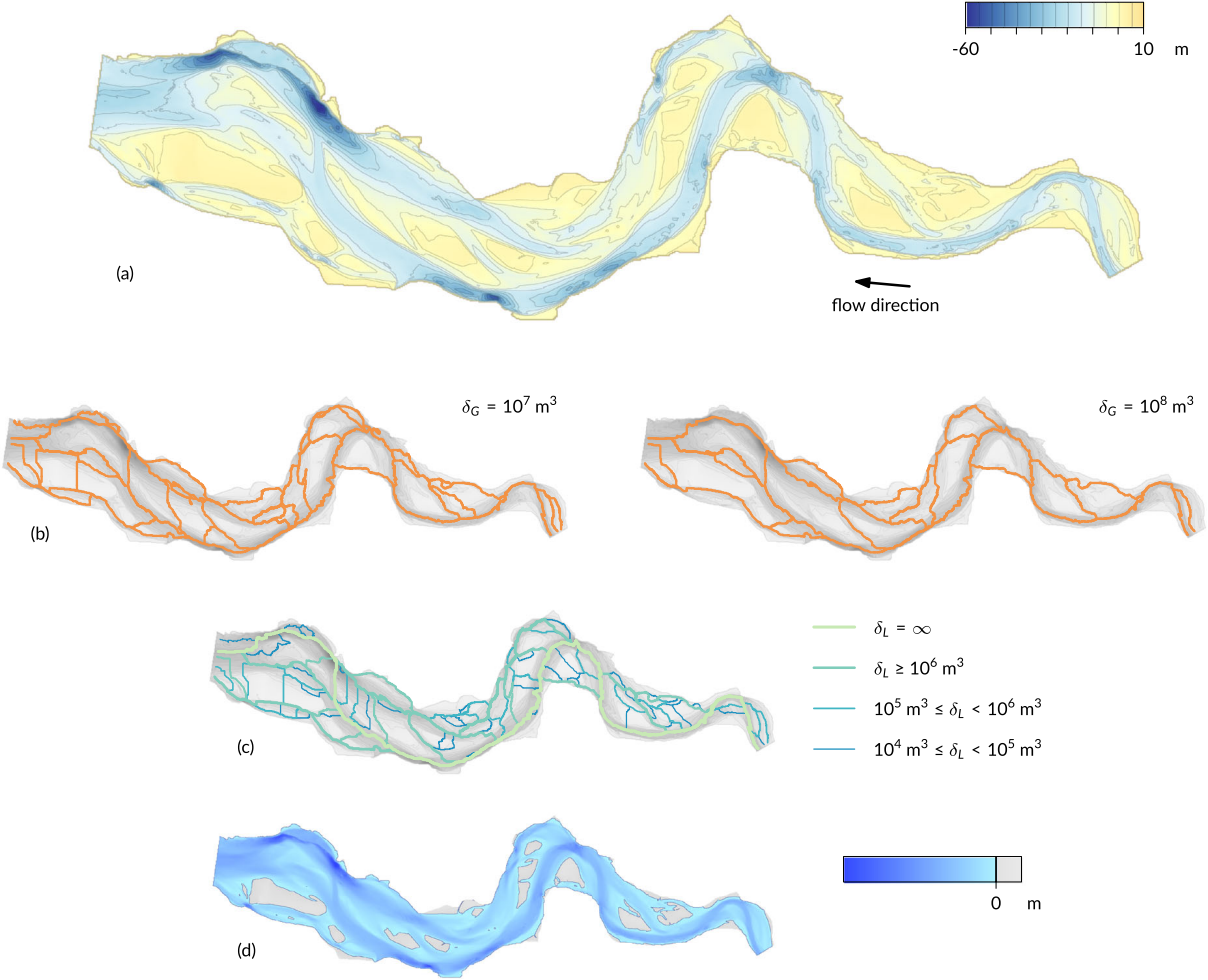
Map of the bathymetry in 2013 (a), two networks created by the striation method with different thresholds for the sand volume *δ*_*G*_ (b), the network by the persistence method where links are colour coded by sand volume *δ*_*L*_ (c), and a sliced morphology map taken at elevation 0 m AMSL (d) [Color figure can be viewed at wileyonlinelibrary.com]

Because both methods select links from the Morse–Smale complex, the positions of individual channels found by the two methods are (nearly) identical (examples in Figure 6). For the striation method, the global sand volume thresholds for distinction of channels were taken at 10^6^, 10^7^, 10^8^ and 10^9^ m^3^, where a larger volume resulted in a sparser network. In the persistence algorithm, the sand volume calculated locally is given as a variable for each network link, and ranges from about 10^4^ to 10^7^ m^3^ (see legend). Regardless of the precise volumes, the positions of the network links with higher volume generally correspond to the larger channels in the estuary (visualised in Figure 6d). The sand volume thresholds in the two methods are quantitatively incomparable, but values can be chosen such that networks of similar detail are created. As explained in Section 5, the network of the striation method is computed separately for each chosen volume threshold for the whole system, while the network of the persistence method is computed once and locally, and can be visualised afterwards for any specified volume threshold.

**FIGURE 7:**
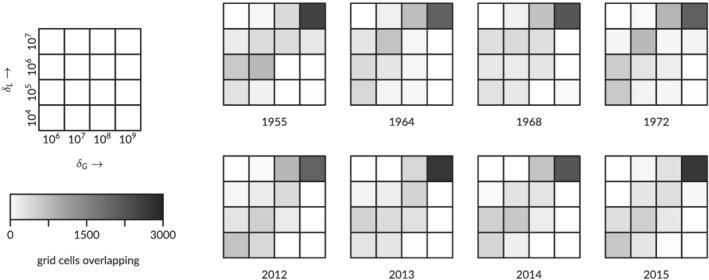
Comparison of the number of corresponding channel positions in four volume classes applicable to the striation method (horizontal, $\delta_{G}=10^{6},\;10^{7},\;10^{8},\;10^{9}$ m^3^) and persistence method (vertical, $\delta_{L}=10^{4},\;10^{5},\;10^{6},\;10^{7}$ m^3^)

The networks of the two methods are compared locally for each grid cell on the maps by intersection, where the volumes from the persistence method are classified for comparison with the networks for given striation volume thresholds. The results are shown for selected years as the number of grid cells for which both methods found a channel (Figure 7), where perfect correspondence would result in non‐zero values only on the diagonal. The correspondence is, however, far from perfect: the highest volume class, identifying the largest channels in the system, has a high overlap but in all lower volume classes the overlap is much smaller. Instead, many channel locations of one method fall into a higher or lower volume class in the other method. For the lowest possible volume (not analysed here), both methods would find the same channels, but the higher volumes of interest visualised here miss all the channels that are detected in one of the methods but not in the other.

**FIGURE 8:**
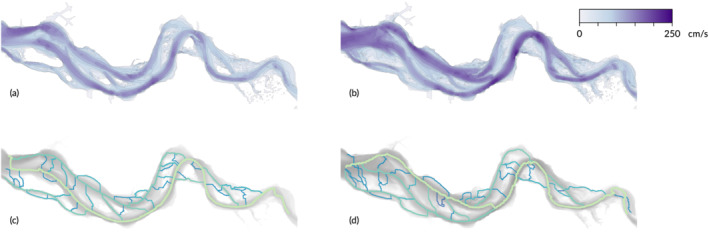
Maps of the flow velocity in cm/s in 2013 for ebb (a) and flood (b), and the networks as created by the persistence method for ebb (c) and flood (d) [Color figure can be viewed at wileyonlinelibrary.com]

Finally, a first comparison between connectivity as determined from bed elevation and connectivity based on flow dynamics generally shows the expected relation between channel morphology and the hydrodynamics in channels. As a test for wider applicability beyond just bed elevation, the persistence method was used on modelled flow fields (Figure 8a,b) to generate networks (Figure 8c,d). Visual comparison of the networks from bathymetry (Figure 6c) shows similarity in the positions of the channels in general, but clear differences in the ebb and flood phase. This is already visible in the flow paths with the largest persistence: in the upstream half of the estuary this path follows the path of the deepest channel, but in the downstream (left) half of the system shifts between the middle and northern channel depending on the tidal phase.

### 3.2 Comparison of properties of the entire network

**FIGURE 9:**
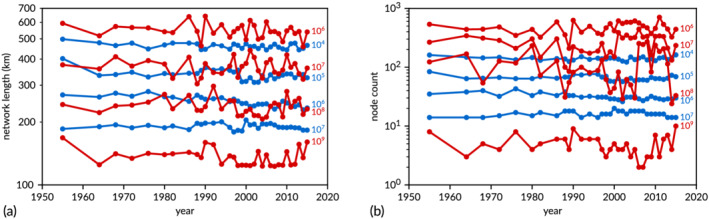
Measures of the whole network. (a) Total length of all network links through time. (b) Number of nodes in the network. Red: striation method, from top to bottom for $\delta_{G}=10^{6},10^{7},10^{8},10^{9}$ m^3^. Blue: persistence method, from top to bottom for $\delta_{L}=10^{4},10^{5},10^{6},10^{7}$ m^3^ [Color figure can be viewed at wileyonlinelibrary.com]

While there is no rigorous method to trace and link the channels through time, the online supplementary movie (Supporting Information) shows that the networks from the two methods show different dynamics over the study period, with larger fluctuations in the striation method than in the persistence method. For each network, we calculated the total length of all links, as a measure of the channel network dimensions for the different threshold volume values. Furthermore, we computed the number of nodes in the networks, as a measure of the network complexity. Both measures are shown in Figure 9. The time series of the total length and the number of nodes show three aspects of the methods.

First, the striation method results in much larger temporal variation than the persistence method, particularly for the lower volume thresholds. The larger variation is not due to some kind of noise, because most rises and falls of the striation network length extend over multiple time steps. To make sense of the volume values, they can be compared with a characteristic control volume of all alluvial material that could be reworked, which is estimated as the product of maximum channel depth (say, 30 m), estuary width (about 5 km) and a characteristic length scale. There are two appropriate length scales. For the global striation method, this is the 65 km length of the estuary centreline between the banks from the Dutch–Belgian border to the mouth. Indeed, the length of the lowest path in both methods is calculated to be about 88 km as it is somewhat more sinuous and follows the edges of the DEM grid. The alluvial volume for this length is about 10^10^ m^3^, which is, as expected, only an order of magnitude larger than the highest striation volume threshold for which a network appears. On the other hand, the characteristic length for the local persistence method is the typical bar length of the bars that separate channels, which is itself related to the estuary width (Leuven et al., 2016). The total alluvial volume for this length is about 10^9^ m^3^, which is two orders of magnitude larger than the highest persistence volume threshold for which a network appears. The reason is that the persistence volume is not calculated from the lowest point upwards, as was the reference alluvial volume, but from the separating saddle point upwards (Figure 4c).

**FIGURE 10:**
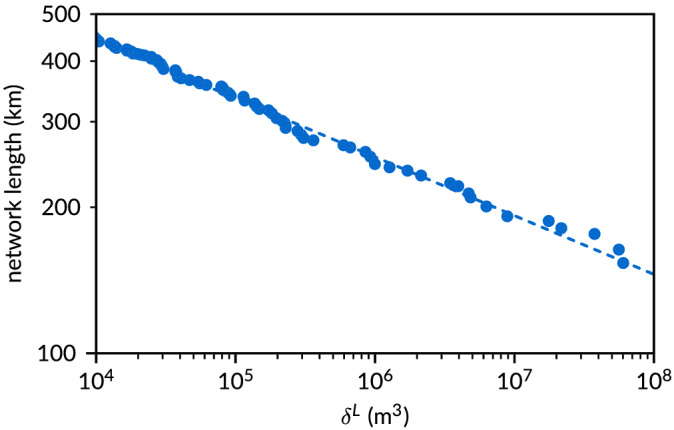
Network scaling for the persistence method applied to the 2013 bathymetry [Color figure can be viewed at wileyonlinelibrary.com]

The persistence method allows a further analysis of the relation between network length, number of links and volume, as each link has been assigned its own specific volume. This shows that the cumulative network length *ℓ* decreases weakly with persistence volume *δ*_*L*_ (Figure 10) by a power law $\ell =1351\delta_{L}^{-0.121}$ with a high regression coefficient $R^{2}=0.995$.

Second, as the threshold volume is reduced by a factor of 10, the total length of the network increases only by about 100–200 km. As the maps (Figure 6) show, the total network length is due to a combination of channel sinuosity, the braiding index and the connections between channels. The more sinuous the channels, the longer is the network length. Likewise, the more parallel channels and the more connecting channels, the longer is the network length. For simplicity, the total network length is here compared with the estuary length. The network is between two and three times larger than the system length for the highest volume thresholds of both methods (lowest lines in Figure 9a) and increases to around eight to ten times larger for the volume threshold that is itself three orders of magnitude smaller.

The time series of number of nodes in the network (Figure 9b) shows the number of bifurcations (or confluences, depending on tidal phase), which is also an indicator of the number of links, or channels, in the network. For comparison, a hypothetical estuary of 25 km length (the Western Scheldt is about 26 km long) with 1–4 km long mid‐channel bars is expected to have around 10 bars and nodes over its entire length, which is more than the number of nodes found by striation for the highest volume threshold, and about correct for persistence. The much higher number for the low volume threshold of the striation method means that a much more detailed network of multiple, relatively short channels is recognised than in the persistence method. The node count increases one order of magnitude for the persistence method but two orders of magnitude for striation, but the increase is a constant factor for persistence and a strongly decreasing factor for striation.

Third, the network lengths for the two intermediate volume thresholds decrease slightly over time, except that of the highest volume thresholds. While the decrease is visible in both methods, there is no significant correlation between the network lengths of the two methods, which is consistent with the difference in algorithms. There are also steps in network length and node count visible that are likely related to large deepening events of the main channel of the Western Scheldt around 1976, 1998 and 2012, which had effects on the entire channel system (studied in van Dijk et al., 2021). The network length and node count for the persistence method show a reduction on the networks with the two intermediate volume thresholds, while the signal of the striation method is too variable for trend recognition.

### 3.3 Channel bifurcation asymmetry

**FIGURE 11:**
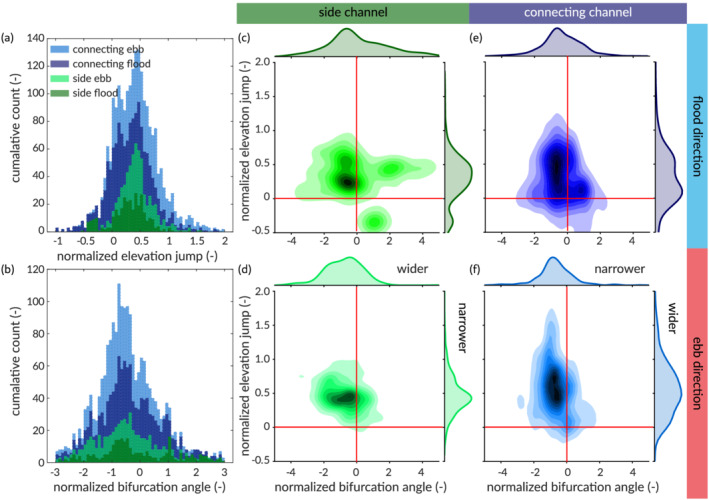
Asymmetry of channel bifurcations calculated from all persistence networks through time, subdivided between bifurcations in the general ebb direction and in the general flood direction, and between large side channels and small connecting channels. (a) Distribution of vertical bifurcation asymmetry: bed elevation differences between the bifurcate normalised with bed elevation (m AMSL) in the upstream channel. (b) Distribution of horizontal bifurcation asymmetry: difference in bifurcate angles with the upstream channel normalised with the sum of the angles. (c–f) Comparison of horizontal and asymmetry for four categories of bifurcations [Color figure can be viewed at wileyonlinelibrary.com]

Bifurcation asymmetry is quantified for all persistence networks together without considering the development through time. As expected, the mean of the distribution of the elevation shows that bifurcations are generally asymmetrical in bed level, independent of their size or direction (Figure 11a). The bifurcations show much variation in horizontal asymmetry but on average the larger bifurcate is more aligned with the undivided channel and the angle of the smaller bifurcate with the main channel is larger (Figure 11b). The joint probability distributions show a correlation between the horizontal and vertical asymmetry (Figure 11c–f), with more spread in the vertical asymmetry of the smaller connecting channels (Figure 11e,f).

## 4 DISCUSSION

### 4.1 Meaning of alluvial connectivity

The first, striation (global) method showed considerable variation through time in its whole‐network properties, whereas the second, persistence (local) method showed much less temporal variation. The dependences of the network length and of the node count on the volume parameter are also different between the methods. This was expected because the volume parameters of the two methods mean different things. First, the striation method calculates the volume from the bottom of the mound that separates two paths, while the persistence volume is calculated from the separating saddle point upwards. Second, the striation volume relates to paths from upstream to downstream boundary, whereas the persistence volume relates to local paths around local highs.

The question is now which method represents volume‐related properties of networks best. The network length and node count variations of up to 200 km in the striation method (Figure 9) seem unwarranted in a system that does not change radically in the observation period. This variation is caused by high sensitivity of the striation path selection to small volume changes somewhere along the path in subsequent time steps. This is relevant for low flow conditions in braided rivers, where the water‐bearing channels will be the lowest paths. However, this sensitivity seems unrealistically high for the estuary studied here and is perhaps also not useful for braided rivers in higher flow conditions. On the other hand, the persistence method considers volumes that need to be eroded for two channels to merge locally, which is closer to the actual morphological process of channel change. The results are consistent between bathymetries that have not changed radically. We conclude that the persistence method is likely of more use in fluvial systems than the striation method.

The concept of connectivity that emerges from the persistence algorithm for network detection on bathymetry is as follows. Alluvial disconnectivity is quantified by the sediment volume that needs to be removed to connect alluvial channel segments. The higher the volume, the greater is the disconnectivity, and the lower the volume, the higher is the connectivity. As such, connectivity is here conceptualised and quantified as the inverse of the sediment volume that needs to be removed in order to connect two channels in an alluvial river or estuary.

As far as we know, a concept of connectivity induced by internal morphological change is novel in the geomorphological literature (for reviews, see Phillips et al., 2015; Wohl et al., 2019). One close example is hydrological connectivity, which is about the physical linkage of water (table 1 in Wohl et al., 2019). Hydrological connectivity is akin to the alluvial connectivity defined here because, in order to gain hydrological connectivity, the alluvial connectivity must be high and the sediment volume to be removed low. Also, sediment connectivity is different, as this is about the degree of linkage that controls sediment fluxes and conveyance through the network. This measure is useful on steeper fluvial systems where fluxes follow existing, long‐lived topographic channels, while triggers for sediment delivery operate on different timescales from sediment transport within the channels, and where linkage, or disconnectivity, is caused by blockage or removal of sediment (e.g., Fryirs, 2013). However, in our case of rivers and estuaries, fluxes and the channel pattern are interacting quickly by the same process of sediment transport, where channel walls consist predominantly of the same cohesionless sediment as transported through the channels. Perhaps the proposed alluvial connectivity is closest to flux connectivity that is related to spatial proximity in a network (Passalacqua, 2017), but alluvial connectivity is about three‐dimensional space, namely volume between network links, rather than travel distance for a flux along or between links in a network.

### 4.2 Network scaling in the Western Scheldt

A surprising outcome was the slow increase of the total network length with sediment volume (Figure 9). This indicates that the network mainly consists of large channels and does not have a fractal structure with a great many smaller channels as found in the radially expanding Wadden Sea tidal basins (Cleveringa & Oost, 1999). Clearly, the estuarine network shows no power law distribution and is not scale invariant. Furthermore, with only one outlet, the estuary is not a delta with subnetworks, so that many meaningful topological measures developed for deltas (Tejedor et al., 2015b) cannot be used here.

The narrow estuarine channel network is dominated by a few large channels separated by large bars, in agreement with bar theories (Leuven et al., 2016). According to fluvial and tidal bar theories, wider and shallower rivers or estuaries lead to a higher number of parallel channels and bars, also expressed as a higher braiding index. Braiding index is, however, an incomplete characterisation of a network, as a braiding index is the same for a few long channels that are parallel over a great length and for many short channels forming a dense network with many nodes.

The number of nodes in the Western Scheldt increases consistently by a factor of about 3, with sediment volume decreasing by a factor of 10 for the persistence method (Figure 9). This scale dependence was also observed in the braided Waimakariri River in New Zealand (fig. 8 in Hiatt et al., 2020). However, this pattern depends on the method as well as on the fluvial system, because the Waimakariri River and other systems in that paper were analysed with the striation method, and the Western Scheldt network shows a much stronger dependence on the striation volume threshold for the large volumes than for the smaller volumes. The cumulative network length decreases weakly but precisely when increasing volume threshold for the persistence method (Figure 10). This is similar to trends, though much less clear, found with the striation method (fig. 10 in Hiatt et al., 2020), except for the Waimakariri River. It is hard to draw conclusions at this point about systems other than the Western Scheldt, and analysis of a set of systems is needed to compare the scale dependence of different systems.

The bifurcation asymmetry in the Western Scheldt network is unexpectedly, consistently mild. Theory suggests that bifurcations are often unstable in low sediment mobility (Bolla Pittaluga et al., 2015), such as in the gravel‐bed Waimarakiri River, and in high mobility, such as in the sand‐bed Jamuna River. The instability implies that relatively more bifurcations should be found in the range of high asymmetry, but that was not the case in the Western Scheldt. Two modelling studies suggest that the reversing tidal currents render tidal bifurcations more stable than fluvial bifurcations (Iwantoro et al., 2020; Jeuken & Wang, 2010) but how this works in a dense network of short, estuarine channels of greatly varying sizes is as yet unknown. The correlation between horizontal and vertical asymmetry points to a causal connection between the angles and depths of the bifurcates that could be similar to the bend effects found in fluvial bifurcations (Kleinhans et al., 2013). These discussion points suggest that the factors determining the different network scaling in rivers, estuaries and deltas require further analysis in relation to the channel and bar pattern‐forming mechanisms.

### 4.3 Outlook

The persistence algorithm opens up the possibilities of rigorous and automated channel network detection in rivers and estuaries on the basis of volume of material between channels, which is a simple and directly measurable characterisation of the amount of morphological work needed to merge the channels. The network properties, topology and derived quantities can be used to compare datasets, numerical models and experiments (Hiatt et al., 2020). This allows expansion of the notion of the delta connectome, which has various forms of connectivity (Passalacqua, 2017), to the estuary connectome or fluvial connectome (see also Hiatt et al., 2021, this issue).

Temporal analyses require, however, that networks can be connected through time, meaning that channels and nodes in different time steps are known to be connected. This would allow analysis of local developments, such as bifurcation morphodynamics, as well as analyses of how natural and human‐induced changes and perturbations are traced through the network. Despite the wealth of data, earlier attempts so far failed especially at the smaller channels, which apparently change too much between bed scans. Especially the smaller‐scale parts of networks change fast, and yet these may be the hotspots of major changes (Howard et al., 1970; Marra et al., 2014; Wohl et al., 2019). However, coupling through time, based on nearest neighbour‐like search rules for connections, so far led to errors particularly at junctions of smaller channels (Marra et al., 2014). At the moment, neither subsequent networks of deltas (Passalacqua, 2017) nor of estuaries (this paper) can be coupled rigorously. It is a challenging open question in computational geometry and topology how to extend this methodology across time to automatically recognise complete spatiotemporal networks; being able to do so would also aid change detection and optimisation of data collection in rivers, estuaries and deltas alike.

The pattern and changeability of the network calculated from the flow field in two moments in the tidal cycle show that, on the one hand, the locations of the channels correspond, but the importance (in terms of persistence) of the channels is temporally much more variable than that of the morphology. This opens up the possibility to compare and analyse both networks calculated from morphology and from the flow field, which are causally connected but changeable at different scales.

The propagation of disturbances through channel networks is of specific relevance. Three distinct signals propagate through networks: namely, the water levels through tidal wave propagation and backwater effects, water flux through fluvial discharge and tidal currents, and sediment transport driven by those currents. In addition, sediment pulses are induced by natural bank collapses, channel migration and avulsion, and by human activity such as dredging and dumping for shipping fairway maintenance and sand extraction (Jeuken & Wang, 2010; van Dijk et al., 2021). How, and how far, such perturbations propagate through the network and contribute to the development of the network through time, are exciting and relevant questions.

## 5 CONCLUSIONS

Two mathematically rigorous algorithms to identify channel networks in bathymetries of fluvial systems were compared conceptually and empirically on a dataset of the Western Scheldt estuary. Both methods distinguish channels on the basis of the alluvial volume between them, but in different ways. The local method, based on persistence, considers the local volume of bars and shoals that need to be eroded in order to join channels, and assigns a specific volume value to each link in the network. This method produces the most consistent results for the data and is conceptually closest to the notion of sediment transport‐generated morphology that causes the pattern of channels and bars.

A new connectivity concept is derived from the local method: alluvial connectivity is the inverse of the sediment volume that needs to be removed in order to connect two channels in an alluvial river or estuary. Hence alluvial disconnectivity is quantified by said sediment volume.

A whole‐network analysis indicated that the persistence‐based network of the Western Scheldt shows a weak power law dependence of cumulative network length and number of network elements with persistence volume but is not scale independent. This property should be compared between many networks as more data are collected. An analysis of the nodes as individual bifurcations shows that there is a persistent asymmetry in the bed elevations and horizontal directions of the channels around the bifurcations, which is unexpected from bifurcation theory. The new fluvial network identification method opens up the possibilities to study the channel networks and their elements through time and to conduct objective comparisons between networks of different systems and of models.

## CONFLICTS OF INTEREST

The authors identify no conflicts of interest.

## Supporting information



ESP_5261_western‐scheldt‐methods‐comparison.mp4

## Data Availability

The supplementary movie ‘western‐scheldt‐methods‐comparison’ shows the time series of bathymetry, of the striation‐based networks for the four chosen volume thresholds, and of the persistence‐based network. The code for the network generation is available at https://github.com/tue-alga/ttga; scripts used for analysis are available from the authors on request. All field data and Simona flow model output from Rijkswaterstaat are publicly available via the service desk at https://www.rijkswaterstaat.nl/zakelijk/open-data and from a variety of web portals. The bathymetry data are available online at Rijkswaterstaat (https://waterinfo-extra.rws.nl/monitoring/morfologie/).
